# Neuroprotective Effects of Polydeoxyribonucleotide in a Murine Model of Cadmium Toxicity

**DOI:** 10.1155/2018/4285694

**Published:** 2018-08-29

**Authors:** Herbert R. Marini, Domenico Puzzolo, Antonio Micali, Elena Bianca Adamo, Natasha Irrera, Antonina Pisani, Giovanni Pallio, Vincenzo Trichilo, Consuelo Malta, Alessandra Bitto, Francesco Squadrito, Domenica Altavilla, Letteria Minutoli

**Affiliations:** ^1^Department of Clinical and Experimental Medicine, University of Messina, Messina, Italy; ^2^Department of Biomedical and Dental Sciences and Morphofunctional Imaging, University of Messina, Messina, Italy

## Abstract

Cadmium (Cd) is a harmful heavy metal, which causes severe brain damage and neurotoxic effects. Polydeoxyribonucleotide (PDRN) stimulates adenosine A_2A_ receptor, thus contrasting several deleterious mechanisms in course of tissue damages. We aimed to investigate the possible neuroprotective effect of PDRN in a murine model of Cd-induced brain toxicity. Male C57 BL/6J mice were treated as follows: vehicle (0.9% NaCl, 1 ml/kg/day), PDRN (8 mg/kg/day), CdCl_2_ (2 mg/kg/day), and CdCl_2_ + PDRN. Animals were tested with the Morris water maze test to assess spatial memory and learning. After 14 days of treatment, brains were processed to evaluate the presence of edema in the cerebral tissue, the expression of mammalian target of rapamycin kinase (mTOR) and brain-derived neurotrophic factor (BDNF), and the morphological behavior of the hippocampal structures. After CdCl_2_ administration, the escape latency was high, protein expression of BDNF was significantly decreased if compared to controls, mTOR levels were higher than normal controls, and brain edema and neuronal damages were evident. The coadministration of CdCl_2_ and PDRN significantly diminished the escape latency, increased BDNF levels, and decreased protein expression of mTOR. Furthermore, brain edema was reduced and the structural organization and the number of neurons, particularly in the CA1 and CA3 hippocampal areas, were improved. In conclusion, a functional, biochemical, and morphological protective effect of PDRN against Cd induced toxicity was demonstrated in mouse brain.

## 1. Introduction

Cadmium (Cd) is an extremely toxic metal with no known necessary function in the human body. It represents serious hazard to human health, as stated by the International Agency for Research on Cancer [1]. Major sources of Cd are food, cigarette smoke, and recharged nickel-cadmium batteries [2]. Foods as cereals, vegetables, nuts and pulses, starchy roots, potatoes, and meat products are the main source of Cd exposure for the nonsmoking population [3].

Several reports studied Cd toxicity in the brain as a whole or in its specific regions. In particular, Cd can experimentally induce neurotoxic effects either *in vitro* or *in vivo*. In fact, damages referred to Cd challenge were observed in cortical and trigeminal neurons [4, 5], in anterior pituitary cells [6], in glioma and neuroblastoma cells [7], and in nerve-glial cell cultures [8]. Neurotoxic effects were also described in neonatal mouse [9] and in adult rat brain [10] and in diabetic rat optic nerve experimentally exposed to Cd [11].

To date, the pathophysiological mechanism of Cd brain toxicity is not completely defined, even if reactive oxygen species (ROS) generation probably plays a crucial role in the detrimental neurotoxic cascade triggered by Cd [12]. Specifically, under these conditions ROS can promote an exaggerated inflammatory response characterized by increased cytokine expression and intercellular adhesion molecule-1 (ICAM-1) upregulation [13], particularly through the activation of nuclear factor-*κ*B (NF-*κ*B) [13]. Moreover, a peculiar role in neurotoxic damage following Cd exposure is played by mitogen-activated protein kinases (MAPKs) [12] able to promote apoptosis [14], as well as by Akt/mammalian target of rapamycin (mTOR) signaling pathway activation, which controls neuron proliferation, growth, and survival [15, 16].

An impaired neurogenesis, with strongly reduced neuronal differentiation and axonogenesis, was observed as a result of Cd-induced neurotoxicity; therefore, neuronal death occurred [17].

So far, in the brain of mammals, an intricate crosstalk underlying both neuroinflammation and neurogenesis provides many possible molecular targets; they might be harmfully impacted by Cd but also, on other side, by suitable therapeutic approaches to counteract Cd-induced neurotoxic effects [17, 18].

Among the neurotrophic factors that support differentiation [19], maturation [20], and survival of neurons [21], the brain-derived neurotrophic factor (BDNF) has neuroprotective effects under adverse conditions, such as glutamatergic stimulation, neuroinflammation, cerebral ischemia, hypoglycemia, and neurotoxicity [22].

Adenosine A_2A_ receptor (ADORA2A) plays a crucial role in many physiological responses and pathological conditions [23]. However, it is still unclear if the role of ADORA2A in the control of neuroprotection is mostly due to the different homeostatic roles of these receptors related with the control of metabolism, of neuron-glial communication, of neuroinflammation, or of the control of action of growth factors [23].

ADORA2A is colocalized with BDNF in the brain, and the functional interaction between ADORA2A stimulation and BDNF action has been proposed [24]. Experimental data indicate that ADORA2A activation is a crucial requisite for the functioning of neurotrophic receptors at synapses. This has been shown for the facilitatory actions of BDNF on synaptic transmission [25, 26] typically on prolonged potentiation at the CA1 area of the hippocampus [27].

Interestingly, a high prevalence of brain function disorders, including cognitive and behavioral impairments, has been associated with mTOR signaling disturbances [28]; in particular, mTOR activation was related to two major signaling pathways, Ras-ERK and PI3K-Akt, that essentially control neuron survival, differentiation, and proliferation in response to extracellular signals [29]. So far, extracellular messengers linked to mTOR activation may involve the adenosine pathway through ADORA2A modulation in response to systemic inflammation [30].

Polydeoxyribonucleotide (PDRN) is the active fraction extracted from trout spermatozoa used for tissue repair [31]; acting through stimulation of ADORA2A, it can well contrast several harmful mechanisms observed in pathological conditions of low tissue perfusion [31–35].

A positive role of PDRN on Cd-induced structural changes of the blood-testis barrier was already demonstrated, suggesting that it may have a positive effect against Cd-induced structural lesions on gametogenesis [36].

In light of this background, PDRN effects in the brain of mice exposed to Cd chloride (CdCl_2_) were investigated to better elucidate the role of this adenosine agonist.

## 2. Materials and Methods

### 2.1. Experimental Protocol

All procedures complied with the standards for care and use of animal subjects indicated by the Guide for the Care and Use of Laboratory Animals (Institute of Laboratory Animal Resources, National Academy of Sciences, Bethesda, MD, USA); they were carried out also in accordance with Directive 2010/63/EU on the protection of animals used for scientific experiments [37, 38]. Fifty-six male adult C57 BL/6J mice (25–30 g), obtained from Charles River Laboratories Italia srl (Calco, Italy), were provided a standard diet ad libitum with free access to tap water under a 12 h light/dark cycle. They were divided into four groups: (i) animals administered with a vehicle solution consisting in 0.9% NaCl (1 ml/kg, ip, daily), indicated as “control + vehicle animals,” (ii) animals administered with PDRN (8 mg/kg, ip daily), indicated as “control + PDRN animals,” (iii) animals challenged with CdCl_2_ plus with the vehicle as above (2 mg/kg, ip, daily), indicated as “CdCl_2_ + vehicle animals,” and (iv) animals challenged with CdCl_2_ (2 mg/kg, ip, daily) and treated with PDRN (8 mg/kg, ip, daily), immediately following CdCl_2_ administration, indicated as “CdCl_2_ + PDRN animals.”

### 2.2. Drugs

CdCl_2_ was purchased from Sigma-Aldrich Srl (Milan, Italy) and diluted to the requested concentration in 0.9% NaCl. PDRN was donated by Mastelli Srl (Sanremo, Italy). All chemicals and reagents were of commercially available reagent grades.

### 2.3. Assessment of Cognitive Performance

To assess spatial memory and learning, animals were tested with the Morris water maze (MWM) test [39]. The test was performed in a round white pool (diameter 80 cm and depth 55 cm). The pool was filled to a depth of 30 cm with water made opaque with white nontoxic water-based tempura paint. Pool temperature was maintained at 22 ± 0.5°C by adding warm water. The escape platform was a 25 cm^2^ plexiglas square, placed in the center of one quadrant of the pool, 15 cm from the pool's edge, and submerged 1 cm below the water surface. The mouse was gently placed in the water pool between the quadrants, facing the wall of the pool changing the order every day during each trial. The mice were given four trial sessions each day for five consecutive days, with an intertrial interval of 15 min. Escape latency time (ELT), that is, the time taken by the animal to move from the starting quadrant to find the hidden platform in the target quadrant, was recorded in each trial, and the average time, expressed in seconds (s), for each day was calculated. If the mouse failed to find the platform within 60 s, it was guided gently onto the platform and allowed to remain there for 20 s. Significant decrease in ELT from that of the first session was considered as successful learning. During all trials, the experimenter always stood in the same position. All trials were performed between 9.00 and 16.00 h in a sound dampened room.

### 2.4. Brain Collection

The experiments lasted 14 days, until the mice were sacrificed with an ip overdose of ketamine and xylazine (100/20 mg/kg, resp.) and then subjected to decapitation. Their skulls were quickly opened, and the brains were extracted on ice and washed with cold phosphate-buffered saline (PBS). The brains of 14 animals for each group were divided as follows: seven brains were used for histological study. From the other seven brains, one half was stored at −80°C for Western blot analysis, and one half was used for edema evaluation.

### 2.5. Evaluation of Brain Edema

To evaluate the extent of edema, brain sections from each group of animals were assayed for water content using wet weight/dry weight. Freshly dissected tissue samples of the hippocampus were weighed on aluminum foil, dried for 24 h at 105°C, and reweighed as previously described [40]. The percentage of water was calculated as follows: water content (%) = (wet weight − dry weight)/wet weight × 100.

### 2.6. Histological Evaluation

Brains were immediately fixed in 4% paraformaldehyde in 0.2 M phosphate buffer solution (PBS), dehydrated in graded ethanol, cleared in xylene, and embedded in paraffin (Paraplast, Supplies SPI, West Chester, PA, USA). 5 *μ*m coronal sections, cut with a RM2125 RT microtome (Leica Instruments, Nußloch, Germany), were cleared with xylene, rehydrated in ethanol, and stained with hematoxylin and eosin (H&E). Histological identification of nervous structures was made according to the atlas of Franklin and Paxinos [41], and the slides were photographed with a Nikon Ci-L (Nikon Instruments, Tokyo, Japan) light microscope; the images were taken with a digital camera Nikon Ds-Ri2 and processed to the final magnification of 800x.

### 2.7. Morphometric Evaluation

Five not serial sections per animal were evaluated for each group. Two experienced investigators, blinded to the experimental group of each animal, independently performed cell counting. The results gave an intraobserver and interobserver variation less than 5%. For hippocampal neurons counts, a region of interest (unit area (UA)) of 0.1 mm^2^ (316 × 316 *μ*m) in both the CA1 and CA3 regions was selected for each section; the cells overlapping the left and the bottom boundaries were counted, whereas the cells that touched the right and top boundaries were not included in the evaluation. Criteria for neurons to be counted were well-defined cytoplasm, clearly visible nucleus, and evident nucleolus.

### 2.8. Determination of Protein Content

Total cellular proteins were extracted in a lysis buffer composed of 25 mM Tris-HCl pH 7.4, 1.0 mM ethylene glycol tetraacetic acid (EGTA), 1.0 mM ethylenediaminetetraacetic acid (EDTA), and 0.5 mM phenyl methylsulphonyl fluoride, added with protease and phosphatase inhibitors (100 mM Na_3_VO_4_, aprotinin, leupeptin, and pepstatin (10 *μ*g/ml each)). After centrifugation of the cell lysate for 15′ at 13000 rpm, the protein concentration was determined from the supernatant by Bio-Rad protein assay (Bio-Rad, Richmond, CA, USA).

### 2.9. Malondialdehyde (MDA) and Glutathione (GSH) Determination

MDA content was determined in all experimental groups with a colorimetric commercial kit (Lipid Peroxidation Assay kit, cat number 437634; Calbiochem-Novabiochem Corp, Darmstadt, Germany), as previously described [42], and expressed in nmol/mg protein. GSH content was also determined in all experimental groups according to the method of Gong et al. [43].

### 2.10. Determination of BDNF and mTOR by Western Blot Analysis

The supernatant was diluted with Laemmli buffer. Protein samples, denatured in reducing buffer (62 mM Tris-HCl pH 6.8, 10% glycerol, 2% SDS, 5% beta-mercaptoethanol, and 0.003% bromophenol blue), were separated by electrophoresis on SDS polyacrylamide gel (6% or 10%), for approximately 1 h. The separated proteins were moved to a PVDF membrane in a transfer buffer (39 mM glycine, 48 mM Tris-HCl (pH 8.3), and 20% methanol) at 200 mA for 1 h. The reaction was blocked with 5% nonfat dry milk in TBS-0.1% Tween-20 for 1 h at room temperature. Membranes were washed three times for 10 min each in TBS-0.1% Tween-20 and incubated with primary antibodies for mTOR (1 : 500 in TBS-0.1% Tween-20; Cell Signaling, Beverly, MA, USA) and BDNF (1 : 1000 in TBS-0.1% Tween-20; Abcam, Cambridge, UK). The following day, the membranes were washed three times for 10 min in TBS-0.1% Tween-20 and were incubated with a specific peroxidase-conjugated secondary antibody (1 : 10.000; KPL, USA) for 1 h at room temperature. After further washings, the membranes were analyzed by enhanced chemiluminescence (KPL, USA). Protein signals were quantified by scanning densitometry with a Bio Image Analysis system (C-DiGit Blot Scanner with Image Studio software), and the results were expressed as relative integrated intensity compared to controls. *α*-Tubulin (Cell Signaling Technology, Beverly, MA, USA) was used to confirm equal protein loading and blotting.

### 2.11. Statistical Analysis

Primary outcome measures were assessment of cognitive performance and neuron morphology. The statistical significance of differences among groups was performed with the ANOVA comparison test, followed by the Bonferroni post hoc test. The MedCalc 12.2.1.0 statistical software (MedCalc Software, Ostend, Belgium) was used. A *p* value ≤0.05 was considered statistically significant. Values are provided as mean ± standard deviation (SD).

## 3. Results

### 3.1. Effects of PDRN on MDA and GSH Content

The levels of MDA were significantly increased in Cd-challenged mice. The coadministration of CdCl_2_ and PDRN significantly decreased the levels of MDA in brains (Table 1). On the contrary, a significant decrease in the activity of GSH was observed in Cd-challenged mice. The treatment with PDRN significantly increased GSH levels in brains of Cd-treated mice (Table 1).

### 3.2. Effects of PDRN on BDNF and mTOR Brain Expression

**Figure 1:**
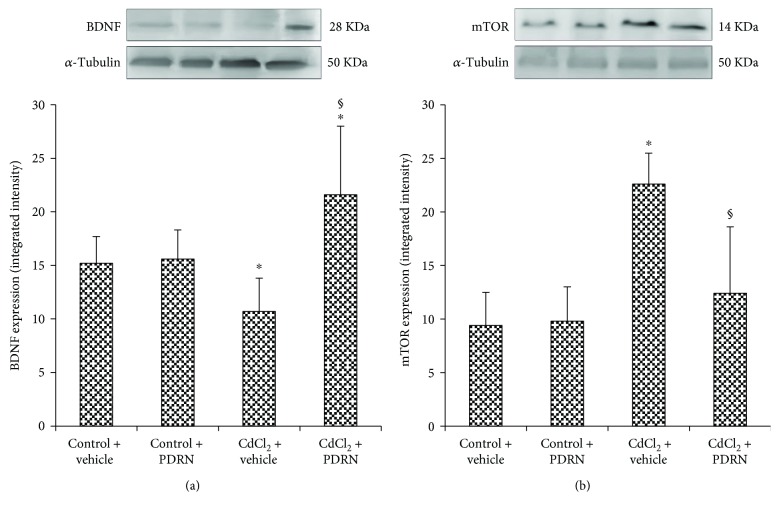
Representative Western blot analysis of BDNF (a) and mTOR (b) in brains of controls and CdCl_2_- (2 mg/kg ip) challenged mice treated with vehicle or PDRN (8 mg/kg ip), respectively. ^∗^*p* < 0.05 versus both controls; ^§^*p* < 0.05 versus CdCl_2_ + vehicle. Bars indicate mean ± SD of 7 experiments.

The expression of BDNF was observed in the brain of control mice treated with vehicle or PDRN (Figure 1(a)). CdCl_2_ caused a marked reduction on BDNF brain expression in mice (Figure 1(a)). Conversely, in mice treated with PDRN, the brain levels of BDNF were significantly higher than in the vehicle-treated CdCl_2_ group (Figure 1(a)).

Low expression of mTOR was observed in the brain of control mice treated with vehicle or PDRN (Figure 1(b)). A higher expression of mTOR was detected in CdCl_2_-treated animals (Figure 1(b)). mTOR expression was significantly reduced after PDRN administration if compared to mice treated with CdCl_2_ alone (Figure 1(b)).

### 3.3. Brain Edema Assessment

**Figure 2:**
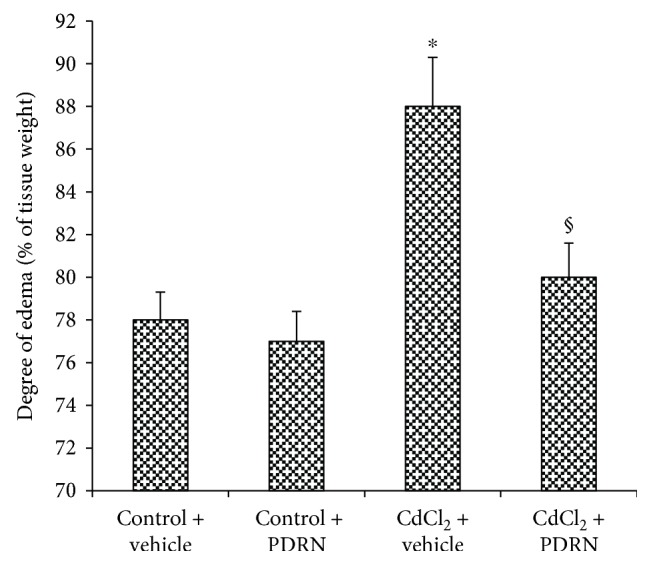
Brain edema evaluated through water content in the hippocampus of controls and CdCl_2_ (2 mg/kg ip) challenged mice treated with vehicle or PDRN (8 mg/kg ip), respectively. ^∗^*p* < 0.05 versus both controls; ^§^*p* < 0.05 versus CdCl_2_ + vehicle. Bars indicate mean ± SD of 7 experiments.

No differences in brain water content were observed in both controls of hippocampal tissue (Figure 2). CdCl_2_ challenge caused brain edema in the mouse hippocampus (Figure 2). PDRN treatment showed a significant reduction of brain edema when compared to CdCl_2_-treated animals (Figure 2).

### 3.4. Administration of PDRN Counteracts CdCl_2_-Induced Neuronal Changes

**Figure 3:**
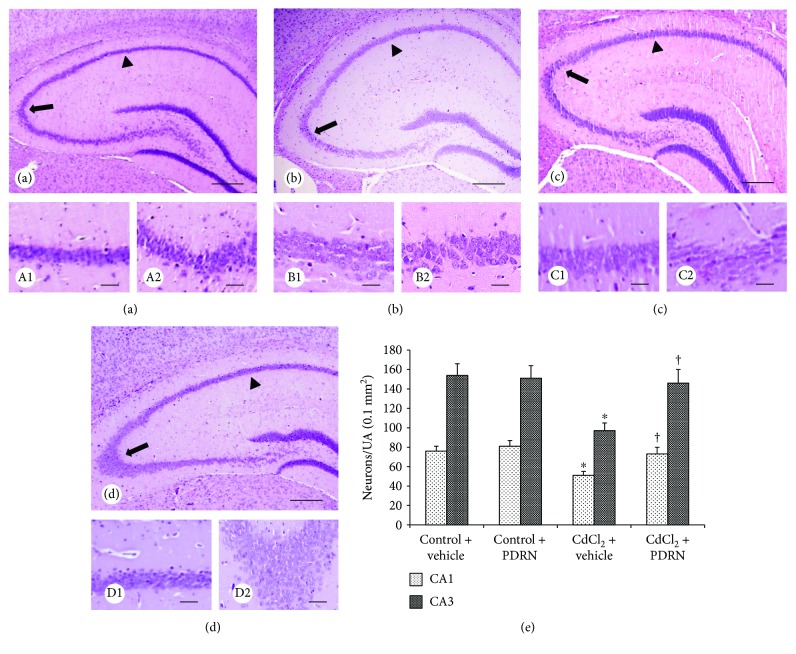
Structural organization of the hippocampus from mice of control plus vehicle (0.9% NaCl, 1 ml/kg/day ip), control plus PDRN (8 mg/kg/day ip), CdCl_2_ (2 mg/kg/day ip) plus vehicle, and CdCl_2_ plus PDRN (HE stain). (a, A1, A2, b, B1, B2) In both control plus vehicle and control plus PDRN-treated mice, the normal morphology of the nervous tissue of the hippocampus, particularly in CA1 (arrowhead) and CA3 (arrow) areas, is evident. (c, C1, C2) In CdCl_2_ plus vehicle-treated mice, neuronal loss and mild edema of the nervous tissue of the hippocampus, particularly in CA1 (arrowhead) and CA3 (arrow) areas, are evident. (d, D1, D2) In CdCl_2_ plus PDRN-treated mice, the hippocampus and CA1 (arrowhead) and CA3 (arrow) areas in particular show a well-preserved neuronal architecture. (e) Quantitative evaluation of neurons in both CA1 and CA3 regions in the different groups of mice. ^∗^*p* < 0.05 versus both controls; ^†^*p* < 0.05 versus CdCl_2_ plus vehicle (scale bar: a, b, c, d = 500 *μ*m; A1, A2, B1, B2, C1, C2, D1, D2 = 50 *μ*m).

In both control groups of mice, CA1 and CA3 hippocampal regions showed normal organization (Figures 3(a), A1, A2, and 3b, B1, B2). In contrast, CA1 and CA3 regions of CdCl_2_-challenged mice showed evident neuronal loss with degenerating pyramidal cells and interstitial edema (Figure 3(c), C1, C2). PDRN administration significantly reduced neuronal morphological changes in both CA1 and CA3 regions (Figure 3(d), D1, D2). The morphometric analysis showed a significant reduction of neurons in both the CA1 and CA3 regions in CdCl_2_-challenged mice, which was normalized when PDRN was coadministered (Figure 3(e)).

### 3.5. PDRN Enhances Cognitive Performance

In both controls, a gradual shortening in ELT was observed along the five-consecutive-day trials (Table 2). CdCl_2_ administration significantly increased ELT when compared with both control groups (Table 2). On the contrary, PDRN administration significantly reduced the time spent by mice to find the platform (Table 2).

## 4. Discussion

Oxidative stress is strongly related to neuroinflammatory mechanisms, so that it is particularly difficult to exert neuroprotective effects on the brain. In addition, glial activation involving astrocytes, microglial cells, and/or reactive mediators and/or growth factors are also hallmarks of inflammatory reaction [44]. This justifies the research strategies that rely on multiple mechanisms involving antiradical scavenging activity and antiapoptotic mechanisms, resulting in increased neuronal proliferation.

Neurotoxic effects may play a key role in the systemic toxic consequences of Cd exposure [45]. Therefore, the mechanism of Cd neurotoxicity should be better clarified, and measures should be taken to reduce Cd exposure in the general population to minimize the risk of adverse human health effects [46]. In this context, we previously demonstrated a positive effect of PDRN, an ADORA2A, which was demonstrated on Cd-induced damages of the blood-testis barrier, suggesting that it should also counteract the role of Cd as an endocrine disruptor [36].

Therefore, our data represent novel findings on the effects of PDRN on the brain, since few information on the molecular pathways involved are currently available.

Indeed, in our *in vivo* experimental model, we observed that mice challenged with CdCl_2_ alone showed a significant increase of MDA and a decrease of GSH; on the contrary, PDRN administration protected mice against oxidative stress, thus confirming the harmful role of Cd in triggering the lipid peroxidation pathways [36].

Furthermore, an increased expression of mTOR in CdCl_2_-challenged mice was demonstrated, whereas PDRN administration significantly reduced mTOR expression. This feature strongly suggests that Cd-induced neuronal toxicity is related to induction of ROS, which, in turn, leads to oxidative stress. In fact, it has been recently shown that Cd induces ROS generation in a time- and concentration-dependent manner in PC12 and SH-SY5Y cells [47], causing apoptosis of neuronal cells, particularly via activation of MAPKs and mTOR signaling pathways [12, 14–16, 47].

Accordingly, we observed that PDRN administration significantly increased BDNF levels in mice. It has been shown that BDNF *in vivo* can rescue different types of neurons from ischemic, traumatic, and toxic brain injury [48]. Recent evidences indicate that the protective effect of BDNF in hippocampal neurons against toxicity is mostly mediated by the PI3K and the Ras/MAPK signaling pathways and involves a long-term change in protein synthesis [29].

Moreover, the role of serine/threonine protein kinase mTOR was also considered [49]. In particular, it has been suggested that mTOR affects the translational control of proteins necessary for the formation and functional maturation of dendritic spines [50]. Moreover, it has been proposed that the neuroprotective effect of BDNF is mediated by autophagy through the PI3K/Akt/mTOR pathway [51]. Our results also show that PDRN administration resulted in a significant reduction of brain edema when compared to the water content of CdCl_2_-treated animals. These effects may be strictly linked with previous explored molecular pathways because it has been suggested that ROS, cytokine overproduction, and neurotrophin reduction are strongly related to brain edema formation in animals challenged with neurotoxic agents [40, 52, 53]. Furthermore, in humans, acute Cd toxicity led to brain intracellular accumulation of the metal with consequent cell dysfunction, blood-brain barrier disruption, and even lethal cerebral edema [54]. It is likely that PDRN can also reinforce the detoxification mechanisms, such as antioxidant systems through the induction of protective macromolecules (heat shock proteins, etc.), production of specific metal inclusion bodies or binding proteins, and biotransformation reactions (methylation, conjugation, etc.) localized in the choroid plexus [17]. Accordingly, ADORA2A was highly expressed in the choroid plexus [55].

The biochemical and molecular patterns correlated very well with the histological analysis. In fact, following CdCl_2_ administration, we observed a significant neuronal loss in both CA1 and CA3 areas, which are susceptible to Cd-induced neurotoxic injury [56]. In contrast, PDRN administration showed significant neuroprotective effects, with a normal number of neurons/UA and structural organization.

Finally, the positive effects of PDRN treatment were also supported in our model by the evidence of a good protection against the behavioral changes that accompanied Cd administration. In fact, PDRN injection significantly improved ELT in mice tested with MWM following CdCl_2_ challenge. This observation could have a strong translational impact considering that Cd causes learning disabilities and hyperactivity in environmentally exposed children [17] and neurological disorders, such as amyotrophic lateral sclerosis [57], Parkinsonism [58], and Parkinson's and Alzheimer's disease [59], in occupationally exposed subjects.

Taken together, our data suggest that adenosine receptor manipulation/modulation is a pertinent avenue of research for novel strategies in order to modulate neuroinflammatory signal into the brain in diseases characterized by impaired immune response induced by toxic agents.

Moreover, in light of our results, we feel that new environmental research on Cd should take aim at the role of neurotoxicity in causing the health effects following Cd exposure.

Both short- and long-duration epidemiological studies are required to determine the optimal doses of antioxidant products and dietary supplements alone and in combination, to provide safe and effective therapeutic strategies against Cd toxicity. In this context, PDRN, an agonist of ADORA2A, might offer a structural model for the production of new analog compounds (cosmeceuticals, nutraceuticals, and/or phytochemicals) that, properly combined with good agricultural practice to minimize Cd contamination in food crops and animals, could also provide a definite strategy to prevent and counteract severe damages in Cd-induced brain toxicity.

## Figures and Tables

**Table 1:** Malondialdehyde (MDA) and glutathione (GSH) content in mice exposed to cadmium chloride (CdCl_2_; 2 mg/kg ip) plus vehicle, as compared to mice exposed to CdCl_2_ (2 mg/kg ip) plus PDRN (8 mg/kg/day ip) or to control mice treated with vehicle or PDRN alone.

Group	MDA (nmol/mg protein)	GSH (*μ*mol/g tissue)
Control + vehicle	0.13 ± 0.04	66 ± 5
Control + PDRN	0.12 ± 0.06	69 ± 6
CdCl_2_ + vehicle	0.81 ± 0.31^a^	47 ± 7^a^
CdCl_2_ + PDRN	0.20 ± 0.09^b^	64 ± 3^b^

All the values are expressed as mean ± SD, *n* = 7 animals for each group. ^a^*p* < 0.05 versus both controls; ^b^*p* < 0.05 versus CdCl_2_ + vehicle.

**Table 2:** Results obtained from the escape latency time (the time to reach the platform in seconds) evaluated with the Morris water maze test in mice exposed to cadmium chloride (CdCl_2_; 2 mg/kg/day ip) plus vehicle, as compared to control mice treated with vehicle or PDRN alone or to mice exposed to CdCl_2_ (2 mg/kg/day ip) plus PDRN (8 mg/kg/day ip).

Group	Day 1	Day 2	Day 3	Day 4	Day 5
Control + vehicle	36 ± 2	31 ± 3 (13.4%)^a^	19 ± 2 (47.3%)^a,b^	14 ± 3 (61.2%)^a,b,c^	11 ± 2 (69.5%)^a,b,c,d^
Control + PDRN	34 ± 3	32 ± 3 (5.9%)	18 ± 4 (47.1%)^b^	15 ± 2 (55.9%)^a,b,c^	12 ± 3 (64.8%)^a,b,c,d^
CdCl_2_ + vehicle	38 ± 3	34 ± 4 (10.5%)^a^	27 ± 3 (28.9%) ^a,b,e^	22 ± 3 (42.1%) ^a,b,c,e^	20 ± 4 (47.3%)^a,b,c,e^
CdCl_2_ + PDRN	33 ± 4	32 ± 4 (3.1%)	20 ± 2 (39.4%) ^b,f^	14 ± 4 (57.6%)^a,b,c,f^	12 ± 2 (63.7%)^a,b,c,f^

All the values are expressed as mean ± SD, *n* = 14 animals for each group. ^a^*p* < 0.05 versus day 1 of the same group; ^b^*p* < 0.05 versus day 2 of the same group; ^c^*p* < 0.05 versus day 3 of the same group; ^d^*p* < 0.05 versus day 4 of the same group; ^e^*p* < 0.05 versus both controls at the same day; ^f^*p* < 0.05 versus CdCl_2_ + vehicle at the same day.

## Data Availability

The data used to support the findings of this study are included within the article.

## Abbreviations

CdCl_2_: Cadmium chloride
ROS: Reactive oxygen species
HE: Hematoxylin and eosin
MAPK: Mitogen-activated protein kinase
MDA: Malondialdehyde
GSH: Glutathione
mTOR: Mammalian target of rapamycin kinase
BDNF: Brain-derived neurotrophic factor
ADORA2A: Adenosine receptor A_2A_
PDRN: Polydeoxyribonucleotide.
