# Corrigendum to “Neuroprotective Effects of Polydeoxyribonucleotide in a Murine Model of Cadmium Toxicity”

**DOI:** 10.1155/2018/9176012

**Published:** 2018-12-13

**Authors:** Herbert R. Marini, Domenico Puzzolo, Antonio Micali, Elena Bianca Adamo, Natasha Irrera, Antonina Pisani, Giovanni Pallio, Vincenzo Trichilo, Consuelo Malta, Francesco Squadrito, Domenica Altavilla, Letteria Minutoli

**Affiliations:** ^1^Department of Clinical and Experimental Medicine, University of Messina, Messina, Italy; ^2^Department of Biomedical and Dental Sciences and Morphofunctional Imaging, University of Messina, Messina, Italy

In the article titled “Neuroprotective Effects of Polydeoxyribonucleotide in a Murine Model of Cadmium Toxicity” [1], Dr. Alessandra Bitto was incorrectly included as an author. The corrected authors' list is shown above. In addition, the Acknowledgments section should be updated as follows:

The investigation was granted by a departmental funding. The authors thank Dr. Alessandra Bitto for the helpful discussions and thank Mister Sebastiano Brunetto of the Department of Biomedical Sciences, University of Messina, for his dedicated technical expertise.
